# Outpatient parenteral antimicrobial therapy for leishmaniasis: 13 years’ experience at a large UK infectious diseases centre

**DOI:** 10.1093/trstmh/trac128

**Published:** 2023-01-05

**Authors:** Vivak Parkash, Nicholas Laundy, Oyewole Christopher Durojaiye

**Affiliations:** York Biomedical Research Institute, Hull York Medical School, University of York, York, UK; South Yorkshire Regional Department of Infection and Tropical Medicine, Sheffield Teaching Hospitals NHS Foundation Trust, Sheffield, UK; Department of Infection, Immunity and Cardiovascular Disease, University of Sheffield, Sheffield, UK; South Yorkshire Regional Department of Infection and Tropical Medicine, Sheffield Teaching Hospitals NHS Foundation Trust, Sheffield, UK; South Yorkshire Regional Department of Infection and Tropical Medicine, Sheffield Teaching Hospitals NHS Foundation Trust, Sheffield, UK; Department of Infection, Immunity and Cardiovascular Disease, University of Sheffield, Sheffield, UK

**Keywords:** cutaneous leishmaniasis, imported tropical infections, *Leishmania*, neglected tropical diseases, OPAT, outpatient parenteral antimicrobial therapy

## Abstract

**Background:**

Leishmaniasis is a neglected tropical disease that is imported by travellers returning to the UK. Given the prolonged therapy required, outpatient treatment has been proven to be cost-effective and safe.

**Methods:**

We describe cases of leishmaniasis treated through outpatient parenteral antimicrobial therapy (OPAT) over a 13-y period (March 2006–September 2018) at a large teaching hospital.

**Results:**

A total of 26 episodes of leishmaniasis were treated successfully, with a mean saving of 14.2 bed-days/episode. Sodium stibogluconate was the most used antileishmanial (92%).

**Conclusions:**

Treatment of chronic infections via OPAT is now commonplace and this approach may be considered for other imported infectious diseases.

## Introduction

The leishmaniases comprise a broad spectrum of diseases caused by the protozoan parasite *Leishmania* and spread by its natural vector, the phlebotomine sand fly. Autochthonous cases are yet to be reported in the UK. Although there are currently no established UK treatment guidelines, treatment is often based on clinician preference, disease manifestation, drug availability and species involved. However, clinical guidance by the Infectious Diseases Society of America is often consulted.^1^ Sodium stibogluconate (SSG), a pentavalent antimonial, and liposomal amphotericin B (LAmB) are frequently used to treat cutaneous leishmaniasis (CL) and LAmB is often used for visceral leishmaniasis (VL). Other antileishmanial agents such as parenteral meglumine antimoniate, pentamidine lesthionate, oral miltefosine and topical paromomycin are rarely used. Hospitalisation is often required due to potential drug toxicity, especially in unstable cases of VL. Current practice avoids the use of thermotherapy and cryotherapy. Outpatient parenteral antimicrobial therapy (OPAT) has facilitated prolonged treatment of infections and has been shown to be a safe, efficacious and cost-effective alternative to hospitalisation.^2^^,^^3^ Although leishmaniasis represents a small proportion of imported infections to the UK, prolonged parenteral therapy and potential drug toxicity represent an appreciable burden.^4^

## Methods

### Patient population and setting

A retrospective analysis was conducted of all episodes of confirmed leishmaniasis that required intravenous (IV) therapy via OPAT at the Department of Infection and Tropical Medicine, Sheffield Teaching Hospitals, Sheffield, UK (a large tertiary referral teaching centre) from January 2006 to September 2018. Patients were identified using an electronic OPAT database. Additional data including demographics, comorbidities, biochemical parameters, antileishmanial regimen, duration of treatment and clinical outcome was gathered from hospital electronic databases. Criteria for diagnosis was based on clinical suspicion with positive polymerase chain reaction (PCR) to detect *Leishmania* DNA from tissue biopsy, visualization of characteristic amastigotes in smears or tissue (histopathology) or positive antileishmanial antibodies detected by serologic tests (direct agglutination test) performed at the UK reference laboratory for tropical diseases. Cases requiring non-parenteral treatment or involving hospitalisation for treatment were excluded.

## Results

We identified 26 episodes of leishmaniasis treated through OPAT during the study period (see Table 1). The mean age was 37.7 y (standard deviation [SD] 12.9) and 69.2% (18/26) were male. *Viannia* subgenus species predominated (42.3% [11/26]), with compatible history of travel to South America. Parasite culture was unsuccessful in all cases. The majority (65.4% [17/26]) of cases were CL, all diagnosed from tissue biopsy, with PCR confirming the organism to at least the subgenus level. A total of 52.9% (9/17) of all CL presented with lower limb involvement.

**Table 1.** Patient baseline characteristics

Characteristics	Values
Age (years), mean±SD (range)	37.7±12.9 (23–68)
Sex, n (%)	
Male	18 (69.23)
Female	8 (30.77)
Comorbidities, n (%)	
HIV	9 (34.62)
Schistosomiasis infection	1 (3.85)
Pancreatitis	1 (3.85)
Hypertension	1 (3.85)
Rheumatoid arthritis	2 (7.69)
Travel history, n (%)	
Argentina	1 (3.85)
Belize	2 (7.69)
Bolivia	2 (7.69)
Brazil	1 (3.85)
Chile	1 (3.85)
Costa Rica	3 (11.54)
Colombia	1 (3.85)
Ecuador	1 (3.85)
Eritrea	9 (34.62)
Ethiopia	1 (3.85)
Guatemala	1 (3.85)
Honduras	1 (3.85)
Libya	1 (3.85)
Nicaragua	1 (3.85)
Pakistan	1 (3.85)
Peru	6 (23.08)
Spain	2 (7.69)
Yemen	1 (3.85)
Therapy, n (%)	
Liposomal amphotericin B	2 (7.69)
Sodium stibogluconate	24 (92.31)
Outcome, n (%)	
Resolution	22 (84.62)
Relapsed	2 (7.69)
Required admission to hospital but subsequently resolved	2 (7.69)
Type of vascular access, n (%)	
Peripherally inserted central catheter	26 (100)
Method of diagnosis, n (%)	
Tissue biopsy and PCR	17 (65.38)
Serology	9 (34.62)
Type of leishmaniasis, n (%)	
Cutaneous	17 (65.38)
Visceral	9 (34.62)
Histopathologic findings, n (%)	
Amastigotes	4 (15.38)
Granulomas	8 (30.77)
Lymphocytic infiltrate	2 (7.69)
Occupational risk factors, n (%)	
Army	1 (3.85)
Bird watcher	1 (3.85)
Expedition leader	1 (3.85)
Wildlife photographer	1 (3.85)
Site affected, n (%)	
Arm(s), cutaneous	3 (11.54)
Face, cutaneous	3 (11.54)
Leg(s), cutaneous	9 (34.62)
Systemic, visceral	9 (34.62)
Torso, cutaneous	2 (7.69)
Adverse events, n (%)	
Acute kidney injury	3 (11.54)
Anaphylaxis	1 (3.85)
Anorexia	1 (3.85)
Confusion	1 (3.85)
Fatigue	2 (7.69)
Low mood	1 (3.85)
Myalgia	1 (3.85)
Myositis	1 (3.85)
Nausea	1 (3.85)
Pancreatitis	7 (26.92)
QTc prolongation	2 (7.69)
Rash	1 (3.85)
Transaminitis	12 (46.15)
Peak ALT pre-treatment (U/l) (n=15), mean±SD	28.2±16.4
Peak ALT post-treatment (U/l) (n= 5), mean±SD	93±60.3
Peak amylase (U/l) (n=15), mean±SD	291.6±406.3
Species, n (%)	
*Leishmania* subgenus (no further speciation)	11 (42.31)
*Leishmania donovani*	2 (7.69)
*Leishmania major*	1 (3.85)
*Leishmania tropica*	1 (3.85)
*Viannia* subgenus (no further speciation)	11 (42.31)
Bed-days saved, mean±SD	14.2±12.5

ALT: alanine aminotransferase.

IV LAmB was used to treat one episode of CL and one episode of VL. Other episodes (16 CL and 8 VL) were treated with IV SSG. The mean duration of patient care (bed-days saved) delivered through OPAT was 14.2 d (SD 12.5; range 0–38) with an estimated saving of £5398.26 per episode of leishmaniasis (1 hospital bed-day costs approximately an additional £385.59 per patient per day)—excluding drug costs and other consumables.^5^ Two cases involving human immunodeficiency virus (HIV) patients with VL treated with SSG relapsed a few months after initial treatment. They were subsequently treated with a prolonged course of alternative agents. Where SSG was used preferentially in LAmB, this was due to renal impairment. Both episodes that required admission involved CL patients who were receiving SSG; one had confusion and the other had a prolonged corrected QT (QTc) interval, both presumed to be treatment associated.

## Discussion

OPAT as IV therapy for leishmaniasis is an effective treatment that results in savings of hospital bed-days. Of isolates in our cohort, 42% were ‘New World’ *Leishmania* species^4^ endemic in the Americas (*Viannia* spp.), which are associated with an increased risk of aggressive disease if left untreated. Most cases imported to the UK are caused by New World species, often requiring more prolonged treatment compared with ‘Old World’ disease, which largely occurs in Asia and Africa.^4^ Old World leishmaniasis may cause clinically benign lesions that can heal spontaneously and therefore may not present to the clinic. IV therapy was used for CL patients where species were either *Viannia* or could not be fully elucidated.

Personal communication with the UK Health Security Agency (UKHSA) suggests that imported infections are not uncommon, although little is known about the causative species or disease manifestations. Between 1996 and 2020, an average of 28.3 cases/y (SD 15.79) were reported to the UKHSA (unpublished data). It is highly likely that leishmaniasis is underreported in the UK since it is not a notifiable disease, unlike many other imported diseases. This highlights the need for improved reporting and accurate diagnostics.

Studies on OPAT for leishmaniasis and other neglected tropical diseases are limited.^3^ OPAT offers the advantage of ongoing clinical monitoring, given antileishmanial agents are associated with adverse events. In our cohort, adverse events were frequent. Biochemical pancreatitis and hepatitis were most common (Table 1) but resolved after cessation of therapy. None of the observed adverse events were severe enough to necessitate discontinuation of treatment. Our study is limited by its relatively small sample and restriction to single-centre experience.

Our cohort included cases treated via OPAT and who would otherwise require inpatient treatment. We hope that our experience alerts clinicians to the utility of OPAT as an alternative to hospitalisation for imported infections requiring prolonged treatment.

## Data Availability

The datasets generated, analysed and supporting the conclusions of this study are available upon reasonable request in writing from VP or OCD.
